# Correlation of [^18^F]SiTATE-PET-tracer with somatostatin receptor expression in meningiomas: an exploratory *ex vivo* histological pilot study

**DOI:** 10.1186/s13550-026-01505-w

**Published:** 2026-09-04

**Authors:** Rieke Gerdes, Jens Schittenhelm, Stephan Singer, Gerald Reischl, Hannes Becker, Marcos Tatagiba, Salvador G. Castaneda-Vega, Christian la Fougère, Benjamin Bender, Nils F. Trautwein

**Affiliations:** 1 Department of Nuclear Medicine and Clinical Molecular Imaging, University Hospital Tuebingen, Otfried-Mueller-Str. 14, Tuebingen, 72076 Germany; 2 Institute of Neuropathology, Department of Pathology and Neuropathology, University Hospital Tuebingen, Calwerstr. 3, Tuebingen, 72076 Germany; 3 Department of General and Molecular Pathology and Pathological Anatomy, University Hospital Tuebingen, Liebermeisterstraße 8, Tuebingen, 72076 Germany; 4 Department of Preclinical Imaging and Radiopharmacy, University Hospital Tuebingen, Roentgenweg 13, Tuebingen, 72076 Germany; 5 Department of Neurosurgery, University Hospital Tuebingen, Hoppe-Seyler-Str. 3, Tuebingen, 72076 Germany; 6 Department of Neuroradiology, University Hospital Tuebingen, Hoppe-Seyler-Str. 3, Tuebingen, 72076 Germany; 7 DFG Cluster of Excellence 2180 ‘Image-Guided and Functional Instructed Tumor Therapy’ (iFIT), University of Tuebingen, Roentgenweg 11, Tuebingen, 72076 Germany; 8 German Cancer Consortium (DKTK), German Cancer Research Center (DKFZ) Partner Site Tuebingen, Auf der Morgenstelle 15, Tuebingen, 72076 Germany; 9 Department of Neurology and Interdisciplinary Neuro-Oncology, University Hospital Tuebingen, Hertie Institute for Clinical Brain Research, Eberhard Karls University Tuebingen, Tuebingen, Baden-Wuerttemberg, 72076 Germany

**Keywords:** PET, PET/MRI, SSTR, [^18^F]SiTATE, Meningioma, Oncology, Immunohistochemical validation

## Abstract

**Background:**

Radioactively labeled somatostatin analogs (SSAs) are used in PET imaging of meningiomas due to the high expression of somatostatin receptor (SSTR) subtype 2. ^18^F-labeled SSTR-tracers offer several economic and logistical advantages over ^68^Ga-labeled tracers, such as a cyclotron-based production, higher activities per synthesis, a lower positron energy resulting in improved spatial resolution, and a longer half-life. Owing to the advantages over the most commonly used ^68^Ga-labeled SSAs, [^18^F]SiTATE - although not yet approved by FDA or EMA - is currently in clinical use. The study compares [^18^F]SiTATE PET uptake with histological SSTR2-expression in meningiomas.

**Results:**

A correlation of SUV values and immunohistochemical SSTR2-expression was performed in 12 patients (42% female, mean age 68.8 ± 7.2 years). This study demonstrates a strong and significant correlation between [^18^F]SiTATE uptake (SUV_mean_) and histological SSTR2-immune-reactivity-score (SSTR2-IRS) values (*r* = 0.80; *p* = 0.002). In addition, a significant difference in [^18^F]SiTATE uptake (SUV_mean_) was observed between low SSTR-expressing lesions (scores 0–1) and high SSTR-expressing lesions (scores 2–3; *p* = 0.006). Furthermore, it was noted that in four cases, the molecular tumor volume (MTV) was underestimated using a threshold value of SUV_min_ 4, presumably caused by a low SUV_max_, while the application of a 50% isocontour threshold led to an underestimation of the MTV in two lesions that showed high SUV_max_ values.

**Conclusions:**

[^18^F]SiTATE PET shows a strong and significant correlation with SSTR2-expression in meningiomas. However, neither of the two threshold methods yielded MTVs comparable to MRI volumes in all cases. Further studies are necessary to identify the conditions under which each threshold value yields the best MTV.

**Supplementary Information:**

The online version contains supplementary material available at https://doi.org/10.1186/s13550-026-01505-w.

## Introduction

Meningiomas are the most common neoplasms of the central nervous system, accounting for more than one third of all primary intracranial tumors [1–4]. Several risk factors have been identified, including exposure to ionizing radiation and genetic syndromes such as neurofibromatosis type 2 (NF2) [1, 2, 4]. Meningiomas arise from meningothelial precursor cells of the arachnoid layer, whose embryological derivation varies depending on their anatomical location, thereby contributing to histological and molecular heterogeneity of meningiomas [2, 4, 5].

Meningiomas are classified into WHO grades 1–3 based on their histology and molecular biomarkers. Following the 2021 WHO update, meningiomas harboring telomerase reverse transcriptase promoter variants (TERTp) and cyclin-dependent kinase inhibitor 2 A/B (CDKN2A/B) homozygous deletions are assigned to WHO grade 3 [2, 6, 7]. TERTp and NF2 variants are associated with a poorer prognosis, whereas CDKN2A/B homozygous deletion is linked to an increased risk of recurrence [7]. Several meningiomas are characterized by an increased expression of SSTR subtype 2 [1, 8, 9].

According to the European Association of Neuro-oncology (EANO) guidelines, diagnostic imaging should rely on Magnetic Resonance Imaging (MRI) and Computed Tomography (CT) in first place. In addition, SSTR-targeted Positron Emission Tomography (PET) can provide valuable complementary diagnostic information, for example for treatment planning in cases of transosseous growth and for management of meningiomas in complex anatomical regions such as the skull base. Furthermore, SSTR-PET can help differentiate between residual tumor and post-therapeutic changes, enabling early treatment and preventing overtreatment [1, 8, 10].

Several SSTR-tracers are available, including gallium-labeled tracers such as [^68^Ga]Ga-DOTA-Tyr3 octreotide ([^68^Ga]Ga-DOTATOC) and [^68^Ga]Ga-DOTA-d-Phe1-Tyr3 octreotide ([^68^Ga]Ga-DOTATATE), as well as fluoride-labeled tracers like [^18^F]SiFAlin-TATE ([^18^F]SiTATE) [8, 11–13]. [^68^Ga]Ga-DOTATOC and [^68^Ga]Ga-DOTATATE, approved by the European Medicines Agency (EMA) and/or the US Food and Drug Administration (FDA) for neuroendocrine tumors, are also frequently used for meningioma imaging and are mentioned in both the EANO Guideline (2021) and the Joint EANM/EANO/RANO/SNMMI Guideline (2024). [^18^F]SiTATE is likewise mentioned in the Joint EANM/EANO/RANO/SNMMI guideline as a possible tracer for meningioma imaging [14–17].

^18^F-labeled SSTR-tracers offer several economic and logistical advantages over ^68^Ga-labeled tracers, such as a cyclotron-based production without the cost-intensive ^68^Ge/^68^Ga generator, higher activities per synthesis, a lower positron energy resulting in improved spatial resolution, and a longer half-life (110 min vs. 68 minutes). These advantages facilitate broader availability of SSTR-PET, while ensuring high image quality with excellent tumor-to-background-ratio [8, 11, 12, 18, 19]. Despite the advantages, [^18^F]SiTATE has not yet been approved by FDA or EMA, which may be attributable to the lack of prospective phase II/III clinical trials, as well as to limited histopathological validation which is currently only available for neuroendocrine neoplasms [20]. The present study therefore aims to provide the first immunohistochemical SSTR2 validation of [^18^F]SiTATE in meningiomas.

## Materials and methods

### Study cohort

**Fig. 1:**
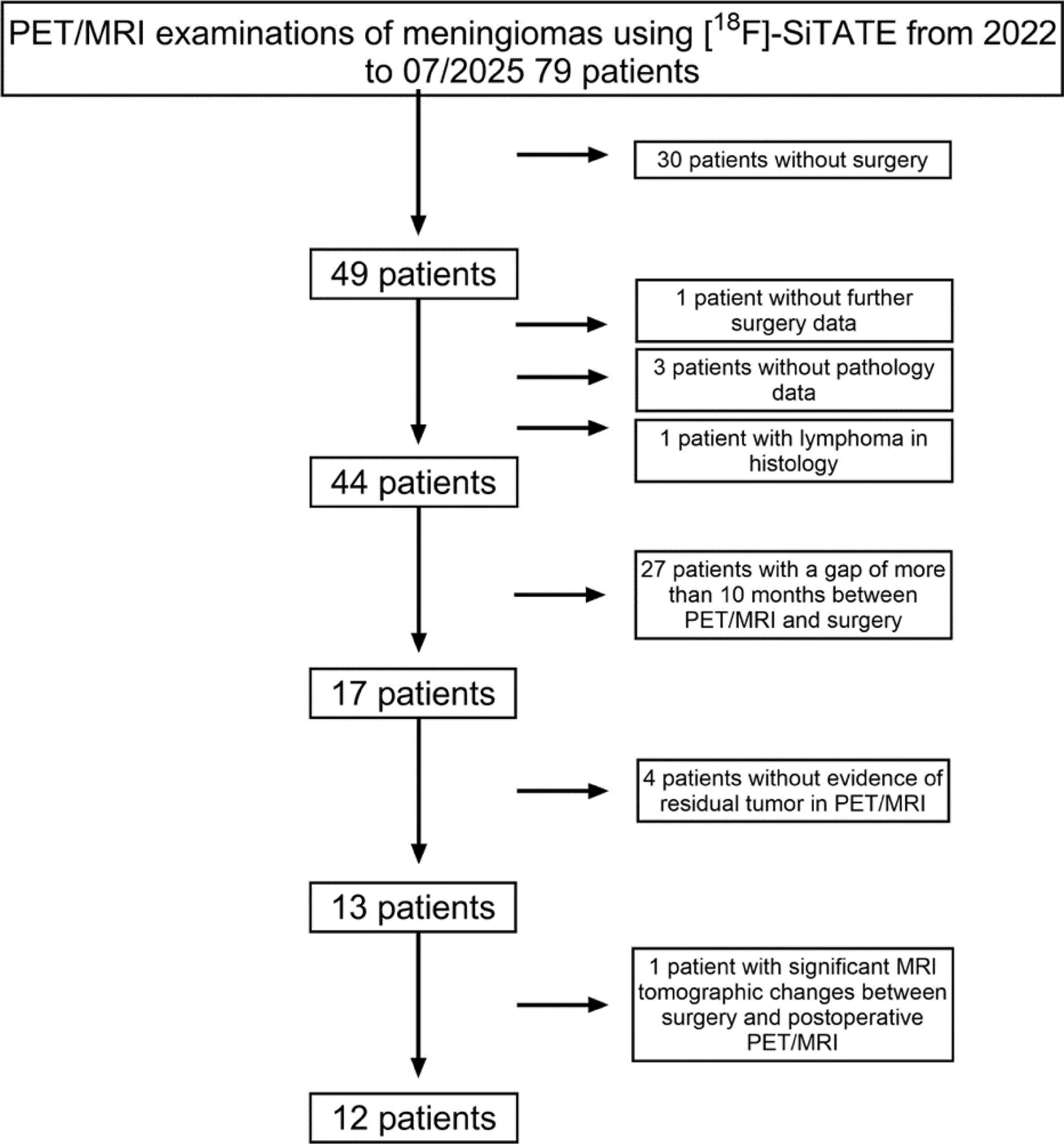
Flowchart of patient selection. The Flowchart illustrating patient selection, inclusion and exclusion criteria and the resulting study cohort of 12 patients

A database search identified 79 patients who underwent [^18^F]SiTATE PET/MRI for suspected, known, or incidentally detected meningioma. Of these, 13 patients had histological confirmation of meningioma in close temporal proximity to the PET/MRI examination and had not received any therapies potentially affecting SSTR-expression, such as radiotherapy, between surgery and PET/MRI. One patient was excluded due to residual tumor with significant morphological changes in the postoperative MRI (Fig. 1). The final study cohort comprised 12 patients. Of these, 7 patients (58%) underwent preoperative [^18^F]SiTATE PET/MRI imaging, followed by surgery within 10 months (3.1 ± 3.4 months). Additional 5 patients with macroscopic residual tumor (42%), but without relevant morphological changes of the residual tumor aspect compared to the preoperative MRI and [^18^F]SiTATE PET/MRI imaging within 4 months after surgery resection (1.8 ± 1.3 months) were included (Supplementary Fig. 1).

General patient data was collected from our database. The study was performed in accordance with the principles of the Declaration of Helsinki. This retrospective study was approved by the Institutional Review Board of the University Hospital of Tuebingen (089/2024BO2). Written informed consent was obtained from all patients in accordance with the provisions of the German Pharmaceuticals Act § 13.2b.

### Tracer synthesis and image acquisition

[^18^F]SiTATE was produced in accordance with Good Manufacturing Practice (GMP) - guidelines using either a TRACERlab MX system (GE Healthcare, Sweden) or a NEPTIS^®^ Perform synthesizer (ORA, Belgium) and cassettes and kits provided by ABX (Germany) [21, 22].

PET/MRI imaging of the head was performed approximately 90 min p.i. (97.3 ± 9.6 min) after injection of [^18^F]SiTATE at a dose of 2.5 MBq/kg BW (2.2 ± 0.4 MBq/kg BW). All examinations were acquired on a 3T PET/MRI-System (Siemens Biograph mMR, Siemens Healthineers, Germany) with an acquisition time of 15 min. Simultaneously acquired MRI imaging was performed with intravenous contrast administration of 0.1 mL Gadovist/kg BW (7.7 ± 2.0 mL Gadovist) and included native and contrast-enhanced 3D T1-weighted sequences; T2-weighted TSE sequences in three planes; axial T2-FLAIR imaging and a diffusion-weighted imaging sequence in most cases (9/12). One patient underwent a whole-body MRI scan covering the area from the head to the proximal thigh due to a rare case of metastatic meningioma.

### Image analysis and Co-registration

Image analysis was performed using Affinity Hybrid Viewer (version 3.0.5, Hermes Medical Solutions, Sweden). [^18^F]SiTATE-uptake of the lesions (SUV_mean_, SUV_max_, and SUV_peak_) and MTV were measured using a volume of interest (VOI) with a threshold of 50% SUV_max_ isocontour and an additional threshold of SUV_min_ 4, as recently proposed for accurate meningioma segmentation [18, 23]. All of the following SUV values are based on a threshold of 50% isocontour, unless otherwise specified. The corresponding values for a threshold of SUV_min_ 4 are shown in Table 1. Semiautomatic VOI segmentation of meningioma/resected meningioma tissue was performed in 10/12 cases (7/12 preoperative, 3/12 postoperative). In 2 postoperative cases a manual adaptation of the VOI placement was performed. For the sake of comparability only postoperative residual meningioma tissue, which was morphological comparable to the resected meningioma and in close spatial proximity to the resected tissue was included in the [^18^F]SiTATE PET semiautomatic VOI segmentation. VOI placements and any required adaptations were performed in consensus with an experienced neuroradiologist (17 years of experience). Particular care was taken to avoid inclusion of postoperative changes in the segmentation and to minimize their potential impact. In case of pituitary involvement, the VOI was changed manually. For blood pool activity assessment, a spherical VOI with a diameter of 4–5 mm was placed in the right internal jugular vein. The tumor-to-blood pool ratio (TBPR) was calculated by dividing SUV_mean_ of the meningioma by SUV_mean_ of the blood pool.

**Table 1:** Descriptive values of various thresholds

SUV_mean_ of meningioma		
50% isocontour	6.8 ± 3.2	
SUV_min_ 4	6.1 ± 1.7	
SUV_max_ of meningioma		
50% isocontour	10.1 ± 4.6	
SUV_min_ 4	10.1 ± 4.6	
SUV_peak_ of meningioma		
50% isocontour	6.8 ± 3.8	
SUV_min_ 4	6.8 ± 3.8	
TBPR		
50% isocontour	6.1 ± 2.8	
SUV_min_ 4	5.6 ± 1.6	
(non)parametric test		
Pearson-Correlation SUV_mean_ and IRS		
50% isocontour (r; [95%-CI]; p)	0.80; [0.42–0.94]; 0.002	
SUV_min_ 4 (r; [95%-CI]; p)	0.797; [0.41–0.94]; 0.002	
Pearson-Correlation TBPR and IRS		
50% isocontour (r; [95%-CI]; p)	0.78; [0.37–0.93]; 0.003	
SUV_min_ 4 (r; [95%-CI]; p)	0.66; [0.14–0.89]; 0.02	
Spearman-Correlation SUV_mean_ and MIB1-proliferation-index		
50% isocontour (r; [95%-CI]; p)	-0.16; [-0.68–0.47]; 0.62	
SUV_min_ 4 (r; [95%-CI]; p)	-0.24; [-0.73–0.40]; 0.44	
SUV 50% isocontour vs. SUV_min_ 4 (t-test)		
Difference; [95%-CI]; p	0.63; [-0.37–1.64]; 0.19	
SUV_mean_ depending on high and low SSTR-expressing groups (t-test)		
50% isocontour (difference; [95%-CI]; p)	5.11; [1.79–8.44]; 0.006	
SUV_min_ 4 (difference; [95%-CI]; p)	2.60; [0.74–4.45]; 0.01	
SUV_mean_ depending on WHO grade (Kruskal-Wallis test)		
50% isocontour (H; p)	1.48; 0.57	
SUV_min_ 4 (H; p)	1.58; 0.56	

In addition, MRI-derived tumor volumes of all lesions larger than 0.5 mL that were classified as meningiomas based on MRI imaging and/or PET/MRI criteria were compared with the MTVs obtained using a 50% isocontour threshold and a fixed threshold of SUV_min_ 4. To avoid disproportionate percentage deviations resulting from minimal absolute volume differences, all meningiomas with an MRI-derived volume of ≤ 0.5 mL were excluded from the analysis. In one patient no evaluation could be performed due to motion artifacts in the MRI images. MTV and MRI volume were considered adequate if the deviation was ≤ 25% or the absolute deviation was ≤ 1 mL. MTVs were classified as overestimated or underestimated if the relative deviation exceeded 25% and the absolute deviation was > 1 mL.

### Immunohistochemistry SSTR2

**Fig. 2:**
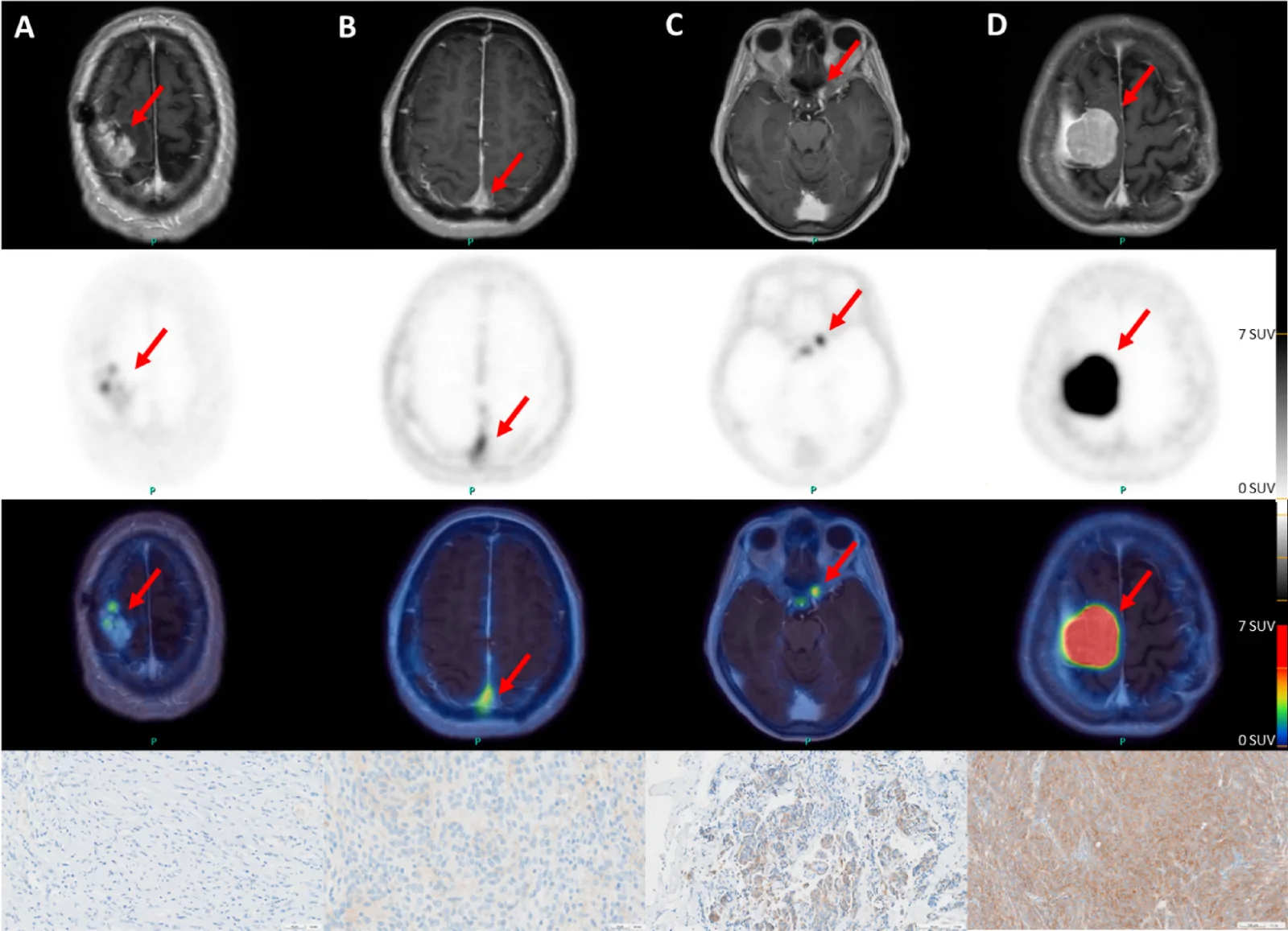
Relationship between MRI, PET, PET/MRI, and histology. Axial contrast-enhanced T1-weighted MRI images (upper row), corresponding PET (upper middle row), fused [^18^F]SiTATE PET/MRI images (lower middle row), and immunohistochemical staining (lower row) are shown. The red arrows indicate the meningioma. The histopathological section shows the expression of somatostatin receptor subtype 2 (SSTR2) according to a score of 0–3. (A) Patient 1: Heterogeneous SSTR-expression of the meningioma in the right superior parietal lobe with a histological score of 0 (SUV_mean_ 2.6). Patient 1 is the only patient in the present cohort with a histological score of 0. In this case, with low and heterogeneous SSTR-expression (SUV_mean_ of 2.6), the score can best be explained by sample bias due to histological confirmation based on meningioma fragments. (B) Patient 7: Low SSTR-expressing meningioma along the superior sagittal sinus with a histological score of 1 (SUV_mean_ 3.4). (C) Patient 10: Moderate SSTR-expression of a meningioma of the left anterior clinoid process with a histological score of 2 (SUV_mean_ 4.5). (D) Patient 2: Strong SSTR-expression of a right parietal meningioma with a histological score of 3 (SUV_mean_ 11.2)

Immunohistochemistry was performed using Roche Ventana Benchmark Ultra strainers (Switzerland), with Cell Conditioner 1 (CC1) used for heat-induced epitope recovery (64 min), SSTR2 (Zytomed, Germany, rabbit, RBK046-05, polyclonal, 1:100, incubation time of 32 min at 37 °C) as the primary IgG antibody and OptiView DAB (Roche, Switzerland) immunohistochemistry detection kit for signal amplification. A modified SSTR2-IRS was used to evaluate immunohistochemistry, which was calculated by multiplying the percentage of positive tumor cells by the intensity factor (0–3) and assigning a score on a scale of 0–300. In addition, a semi-quantitative scoring system (scores 0–3) based on Volante et al. [24], originally established for neuroendocrine tumors, was used. A score 0 indicating no immunostaining, score 1 only cytoplasmic staining, score 2 membranous staining < 50%, and score 3 membranous staining > 50%. Scores 0 and 1 were classified as low SSTR-expression, and scores 2 and 3 as high SSTR-expression (Fig. 2).

### Statistical analysis

The distribution of the data was assessed for normality using the Shapiro-Wilk-Test. For normally distributed variables Pearson correlation was used to analyze the association between SUV_mean_ vs. SSTR2-IRS and TBPR vs. SSTR2-IRS. For non-normally distributed variables, Spearman correlation was applied to assess the association between SSTR2-IRS vs. MIB1-proliferation-index. The t-test was applied to compare the SUV values in two groups (normally distributed) and Kruskal-Wallis test to compare more than two groups (non-normally distributed). We addressed potential limitations related to normality assumptions in the small cohort by performing additional non-parametric statistical analyses, which are provided in the supplementary material (Supplementary Table 2). All statistical tests were conducted at a significance level of α = 0.05. Arithmetic mean and standard deviation were used for descriptive statistics. GraphPad Prism (Version 9.4.1, GraphPad Software, USA) was used for statistical analysis and figures.

## Results

### Study population

Of the 12 included patients (42% female, mean age 68.8 ± 7.2 years), 7 (58%) underwent preoperative [^18^F]SiTATE PET/MRI imaging, whereas 5 patients (42%) underwent postoperative [^18^F]SiTATE PET/MRI examination. WHO grades 1, 2 and 3 were present in 33%, 58% and 8% of patients, respectively. Molecular genetic analysis was available in 6 patients (50%), with one patient exhibiting a heterogeneous deletion of CDKN2A and CDKN2B, as well as NF mutation (WHO grade 2). The mean MIB1-proliferation-index was 11.3 ± 14.7. 3 patients (25%) had not received any tumor-specific therapy prior to PET/MRI, whereas 4 patients (33%) had undergone prior therapies beyond surgery. Meningioma recurrence was observed in 6 patients (50%). Detailed patient characteristics are summarized in Table 2.

**Table 2:** Characteristics

Patient count (*n*)	12
Age (y)	68.8 ± 7.2
Sex - n (%)	
Male	7 (58)
Female	5 (42)
PET/MRI and surgery - n (%)	
PET/MRI before surgery	7 (58)
Time between PET/MRI and surgery (months), mean ± SD	3.1 ± 3.4
PET/MRI after surgery	5 (42)
Time between surgery and PET/MRI (months), mean ± SD	1.8 ± 1.3
Relapse - n (%)	6 (50)
Metastases - n (%)	1 (8)
Therapy before PET/MRI - n (%)	
None	3 (25)
Surgery	9 (75)
Radiation	4 (33)
Systemic therapy (Everolimus, Octreotide, Bevacizumab)	2 (17)
PRRT	2 (17)
WHO-Grade - n (%)	
1	4 (33)
2	7 (58)
3	1 (8)
Genetic analysis - n (%)	6 (50)
Heterozygous deletion of CDKN2A and CDKN2B	1 (8)
NF-mutation	1 (8)
MIB1-proliferation-index (%), mean ± SD	11.3 ± 14.7

### PET imaging analysis

**Fig. 3:**
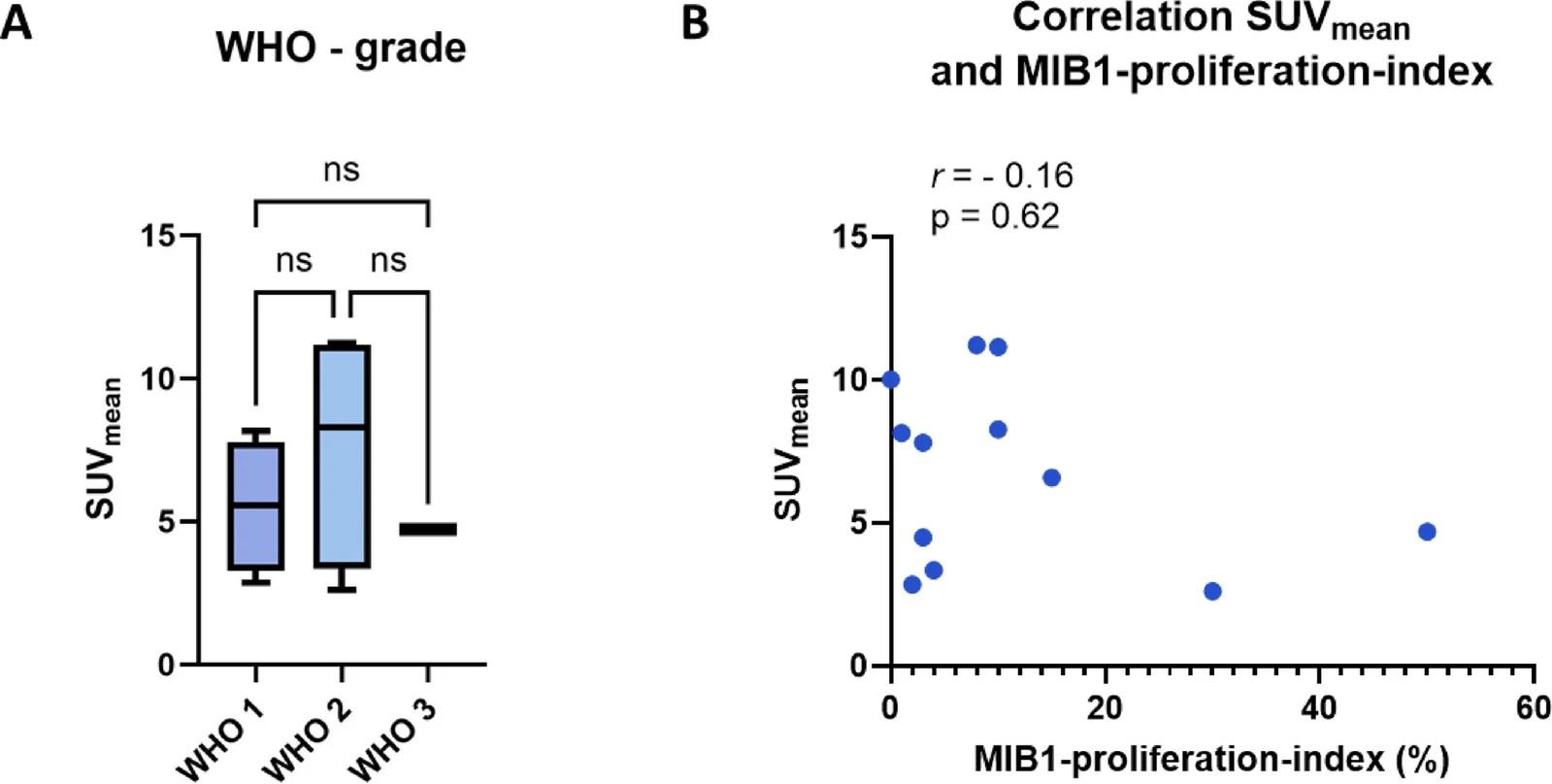
Correlation between tumor grade, MIB1-proliferation-index and SUV_mean_. (**A**) Distribution of mean standard uptake value (SUV_mean_) according to WHO grade (grade 1–3). The box plots represent the range (minimum to maximum) of SUV_mean_ values. No statistically significant differences were found between the groups (*p* = 0.57). (**B**) Scatter plot illustrating the correlation between SUV_mean_ and the MIB-1-proliferation-index (%). No significant correlation between SUV_mean_ and proliferation rate was found

PET images were analyzed with focus on the tracer uptake in meningiomas, blood pool activity and MTV. All meningiomas showed an increased SSTR-expression according to the PET measurements, with an average uptake of SUV_mean_ (6.8 ± 3.2), SUV_max_ (10.1 ± 4.6) and SUV_peak_ (6.8 ± 3.8), as summarized in Table 1. Blood pool SUV was measured in the right internal jugular vein (1.1 ± 0.2) to calculate the TBPR (6.1 ± 2.8). No significant difference in SUV_mean_ was observed across WHO grades (*p* = 0.57; Fig. 3A). Furthermore, no significant correlation was found between SUV_mean_ and the MIB1-proliferation-index (*r* = − 0.16; *p* = 0.62; Fig. 3B). Corresponding uptake parameters calculated using a fixed threshold of SUV_min_ 4 are presented in Table 1. No significant difference was observed between SUV_mean_ values obtained using different segmentation thresholds (SUV_min_ 4: 6.1 ± 1.7; 50% isocontour: 6.8 ± 3.2; *p* = 0.19).

### Immunohistochemical SSTR2-expression and SUV-uptakes

**Fig. 4:**
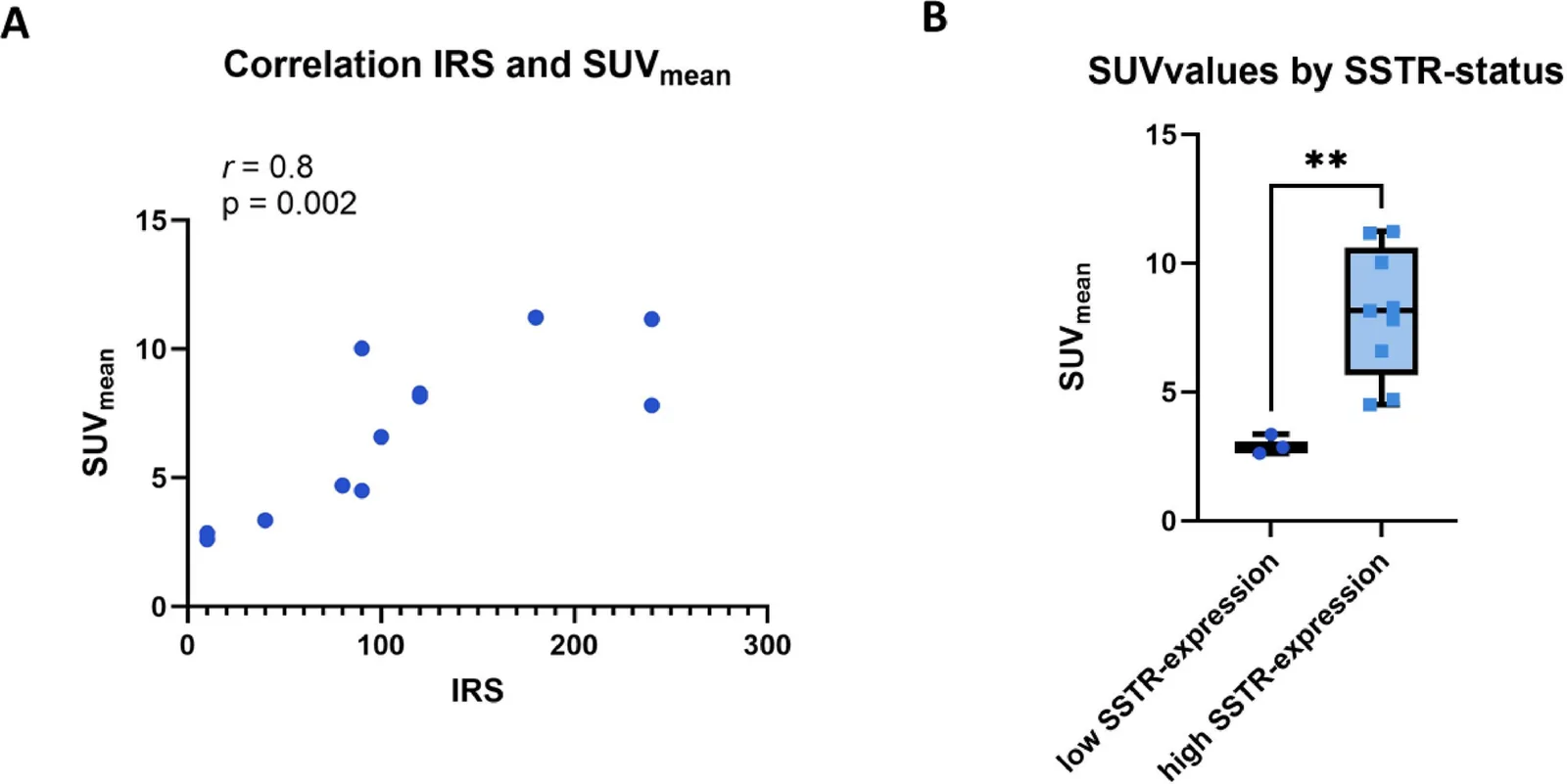
Correlation between IRS and SUV_mean_ and comparison of SUV values according to SSTR-status. (**A**) Scatter plot showing the correlation between the immunoreactive score (IRS) and the mean standard uptake value (SUV_mean_). A statistically significant correlation was observed. (**B**) Box plot illustrating SUV values according to SSTR-status. The boxes represent the median and range (minimum to maximum). SUV values differed significantly between the low and high SSTR-expressing groups (*p* = 0.006)

A significant positive correlation was shown between SUV_mean_ vs. SSTR2-IRS and TBPR vs. SSTR2-IRS (*r* = 0.80 *p* = 0.002; *r* = 0.78 *p* = 0.003) (Fig. 4A). In addition, SUV_mean_ differed significantly between high SSTR-expressing lesions (scores 2–3, mean 8.1) and low SSTR-expressing lesions (scores 0–1, mean 2.9; *p* = 0.006) based on the semi-quantitative scoring system (Fig. 4B).

### Comparison of tumor volume with different threshold values

**Fig. 5:**
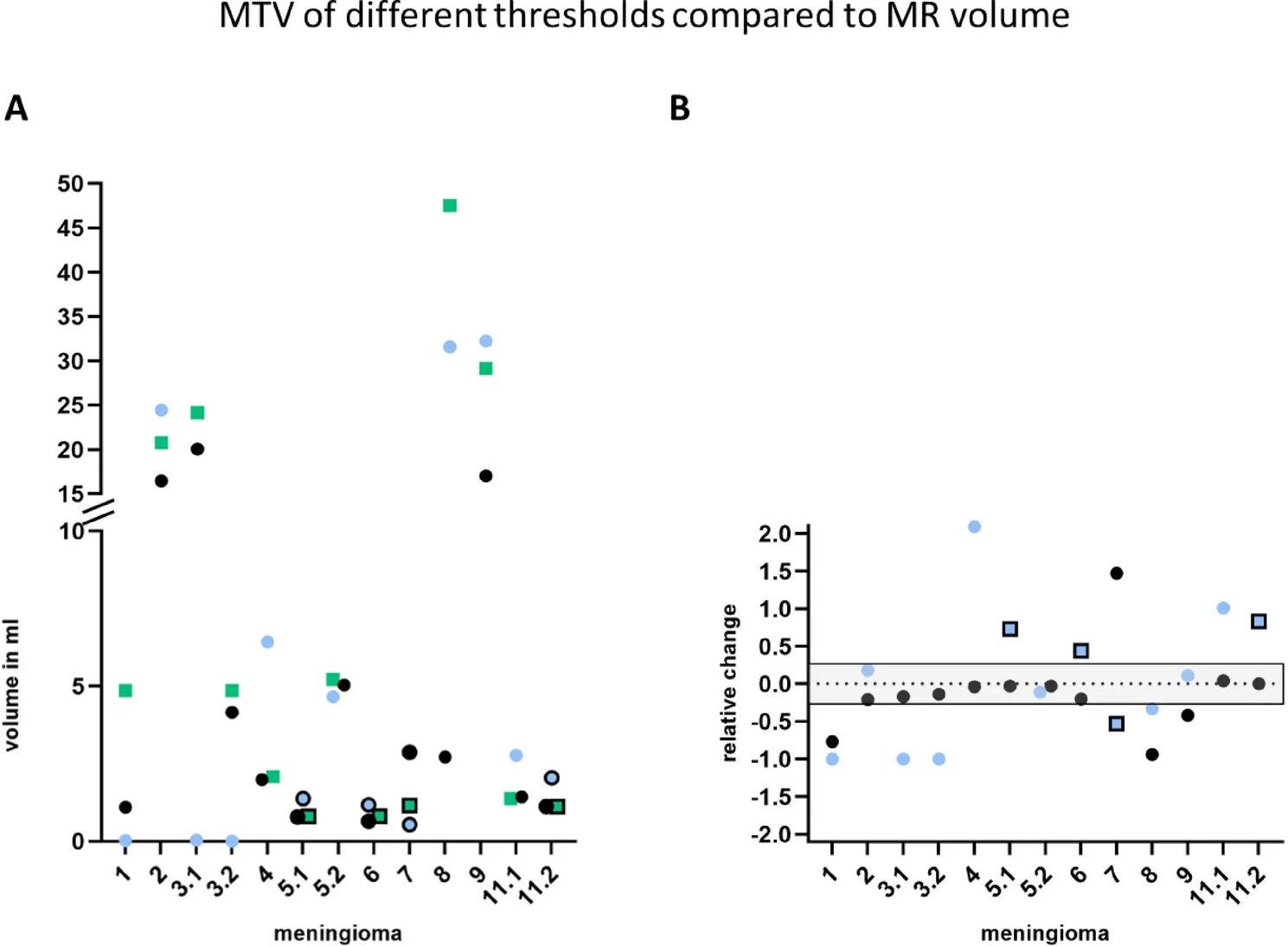
Comparison of MRI derived meningioma volume and MTV (**A**) Meningioma volumes measured using MRI (reference standard) are shown alongside metabolic tumor volume (MTV) values determined using two different PET-based thresholds. The green squares represent MRI derived tumor volumes, blue circles represent MTV values calculated using an SUV_min_ threshold of 4, and black circles represent MTV values calculated using a threshold of 50%. The framed symbols indicate four patients in whom the MTV based on the SUV_min_ threshold of 4 was considered comparable to the MRI volume despite a deviation of more than 25%, as the absolute volume deviation was ≤ 1 mL. (**B**) Percentage deviation of MTV from MRI-based tumor volume for both thresholds. The gray shaded area shows the predefined acceptable deviation range of ± 25% from the MRI volume (dashed reference line)

A total of 23 lesions were classified as meningiomas or residual meningiomas after resection according to MRI and/or PET criteria. Of note, five meningiomas were missed on MRI and were identified only by the additional PET information (21.7%). These lesions were excluded from further analysis due to the absence of MRI-derived volumes. After excluding another five meningiomas with an MRI volume of ≤ 0.5 mL, volumetric analysis was performed on the remaining 13 lesions. Using a 50% isocontour threshold, 9 MTVs (69.2%) were considered comparable to MRI-derived volumes, with an average absolute deviation of 1.1 mL. In contrast, segmentation using a fixed threshold value of SUV_min_ 4 resulted in 7 adequately comparable MTVs (53.8%, average deviation of 1.4 mL). Among these, 4 MTVs (30.8%) showed a relative deviation > 25% but an absolute deviation ≤ 1 mL (Fig. 5; Table 3) and were therefore classified as comparable according to the predefined criteria.

**Table 3:** Volume

Meningiomas - *n* (%)	23 (100)
False MRI negative - n (%)	5 (21.7)
Volume *n* = 18 (mL), mean ± SD	8.1 ± 13.4
Volume ≤ 0.5 mL - n (%)	5 (21.7)
Volume *n* = 13 (mL), mean ± SD	11.1 ± 14.8
Threshold 50%	
PET volume ± 25% of MRI volume - n (%)	9 (69.2)
Difference (mL), mean ± SD	1.1 ± 1.8
PET volume > 25% - n (%)	1 (7.7)
Difference (mL), mean ± SD	1.7 ± 0.0
PET volume < 25% - n (%)	3 (23.1)
Difference (mL), mean ± SD	20.2 ± 21.7
Threshold SUV_min_ 4	
PET volume similar to MRI volume - n (%)	7 (53.8)
PET volume ± 25% of MRI volume - n (%)	3 (23.1)
Difference (mL), mean ± SD	2.5 ± 1.7
PET volume difference ≤ 1 mL	4 (30.8)
Difference (mL), mean ± SD	0.6 ± 0.2
PET volume > 25% - n (%)	2 (15.4)
Difference (mL), mean ± SD	2.9 ± 2.1
PET volume < 25% - n (%)	4 (30.8)
Difference (mL), mean ± SD	12.4 ± 9.4

Further analysis of MTVs that were more than 25% smaller than the corresponding MRI-derived volumes revealed that underestimation using a SUV_min_ 4 threshold occurred predominately in meningiomas with a low SUV_max_ value (*n* = 4, patient 1, 3 and 7 in Supplementary Table 1). Three of these four lesions were histologically confirmed, including all three low SSTR-expressing meningioma lesions. The MTV of the fourth meningioma (SUV_max_ of 3.3) could not be delineated using the SUV_min_ 4 threshold. In addition, two cases with high SUV_max_ demonstrated substantially smaller MTVs, when segmented using a threshold of 50% isocontour (patient 8 and 9 in Supplementary Table 1). In both patients, MTVs derived using a SUV_min_ 4 threshold were more consistent with the MRI volumes than those derived using a threshold of 50%. Patient 1 (see Supplementary Tables 1 and Fig. 2A) showed heterogeneous SSTR-expression and an underestimate MTV regardless of the threshold. However, with a small SUV_max_ (4.1), the MTV with a threshold of 50% was closer to the MRI volume (percentage difference 77.4 vs. 99.6%).

## Discussion

The SSTR-tracers [^68^Ga]Ga-DOTATOC and [^68^Ga]Ga-DOTATATE, which are approved by EMA and/or FDA for imaging of neuroendocrine tumors, are also frequently used for PET imaging of meningiomas [14–17]. Despite the potential advantage of the ^18^F-labeled SSTR-tracers, no such tracer has yet received approval by EMA or FDA, likely due to the still limited data available on the clinical and histological validation. To address this gap, we performed SSTR2 antibody validation of [^18^F]SiTATE uptake in 12 histologically confirmed meningiomas.

We demonstrated a strong positive and statistically significant correlation between tracer uptake (represented by SUV_mean_ and TBPR) and immunohistochemical SSTR2-expression (represented by SSTR2-IRS). In addition, tracer uptake differed significantly between the immunohistochemically high and low SSTR-expressing group, with higher SUV_mean_ values in the high SSTR-expressing group. When comparing our findings with previously published data, differences in immunohistochemical staining evaluation, scoring and different thresholds must be considered. Nevertheless, our results are consistent with the recently published significant correlation between [^18^F]SiTATE tracer uptake (SUV_mean_ and TLR) and histological SSTR2-expression (H-score) in 47 patients with neuroendocrine tumors (*p* < 0.0001) [20].

Furthermore, our results are in line with previously reported correlations between [^68^Ga]Ga-DOTA-SSA and immunohistological SSTR2-expression in meningiomas. Rachinger et al. demonstrated a positive correlation between SSTR2-expression and [^68^Ga]Ga-DOTATATE uptake represented by SUV_max_ (*p* = 0.008) in 21 patients/115 samples [25], Ren et al. showed significant higher SUV_max_ values in tumors positive for SSTR2A-expression in 80 patients using [^68^Ga]Ga-DOTATATE PET/CT (*p* = 0.023) [26] and Iglseder et al. showed a significant correlation in 18 samples between [^68^Ga]Ga-DOTATOC tracer uptake in PET/CT (represented by SUV_max_) and IRS for SSTR2A (r^2^ = 0.669, *p* < 0.001), SSTR2B (r^2^ = 0.393, *p* = 0.001), and SSTR5 (r^2^ = 0.235, *p* = 0.012) [27].

Our results demonstrate no significant difference in SUV values in the WHO groups, in line with previously reported heterogenous data on [^68^Ga]Ga-DOTA-SSA tracers and tumor growth rate across WHO groups [25, 26, 28].

With regard to potential treatment planning, we compared the MTV at a threshold of 50% isocontour and fixed threshold value of SUV_min_ 4 of all meningiomas classified according to PET and/or MRI criteria with the MRI-derived volumes. Notably, approximately 22% (5/23) of meningiomas were not detected on MRI imaging but seen on additional PET information. This proportion exceeds previously reported rates of meningiomas overlooked in MRI imaging. Two studies showed that 8% of meningiomas (175/190 and 103/112) were detected only by using [^68^Ga]Ga-DOTATOC PET/CT [29] and [^68^Ga]Ga-DOTATOC PET/MRI information [30] compared to MRI information alone. This discrepancy is likely attributable to differences in patient cohorts, as the present study included a high proportion of complex, frequently preoperated meningiomas.

In our cohort, four MTVs using a threshold of SUV_min_ 4 were markedly smaller than the corresponding MRI volume, presumably due to low SUV_max_ values. In one case the MTV could not be delineated using a threshold value of 4 owing to SUV_max_ < 4, despite an MRI volume of 4.9 mL. This limitation of a fixed SUV threshold has been described by Mansour et al. with the proposed threshold SUV_min_ 4 who reported similar findings in 17 cases using [^18^F]SiTATE, predominantly involving small lesions [18].

Excluding all meningiomas ≤ 0.5 mL, MTVs using a threshold of 50% isocontour showed better agreement with MRI volumes than those derived using a threshold of SUV_min_ 4 (comparable MTV and MRI volume in 69% vs. 54% cases). However, in two cases with high SUV_max_ values (15.1 and 19.1) MTVs segmented using a 50% isocontour underestimated the volume. Therefore, neither threshold approach resulted in consistently comparable MTVs in all cases.

Based on our limited cohort, a SUV_min_ 4 threshold may be more suitable for lesions with high and focal SUV_max_ values, whereas using threshold with an isocontour of 50% could be more appropriate for meningiomas with lower overall tracer uptake since absolute SUV thresholds may result in undersegmentation or even failure to identify lesions with low uptake. However, this hypothesis requires validation in a prospective study with a larger cohort and ideally with a tumor volume determined postoperatively and ex vivo.

### Limitation

The main limitation of this study is the small and heterogeneous patient cohort with pre- and postoperative SSTR-PET/MRI. Especially the correlation of postoperative SSTR-imaging with IHC findings may be a source of a bias. To reduce this limitation only morphologically comparable tissue areas between pre- and postoperative imaging were included. Nevertheless, postoperative image-histology correlation remains an approximation. An additional limitation is the time interval between PET/MRI and surgery, which may have impact on potential changes in SSTR-expression. Nevertheless, patients receiving SSTR-expression modulating treatment between imaging and surgery were excluded to mitigate this effect. Furthermore, sampling bias may have occurred because histological analysis was performed on meningioma fragments, whereas tracer uptake was assessed for the entire tumor in most cases, which cannot be avoided due to infeasibility of whole-tumor staining. In addition, no standardized SSTR2 immunohistochemistry scoring system exists for meningiomas, necessitating the use of a system originally developed for neuroendocrine tumors.

The comparison of PET-derived MTV with MRI-derived tumor volume represents another limitation, as MRI-based volumetry is subject to delineation bias and interobserver variability and therefore cannot be considered as a true gold standard. Furthermore, only two of several existing segmentation approaches were evaluated, with no universally accepted threshold available. Overall, the evaluated segmentation approach remains exploratory and requires further validation in larger studies.

## Conclusion

The study provides data on the first immunohistochemical ex vivo validation of [^18^F]SiTATE tracer in meningiomas. Our data revealed a strong, positive and significant correlation between tracer uptake and SSTR2-expression. Furthermore, both thresholds used (50% isocontour and SUV_min_ 4) for calculating the MTV were not ideal. Further studies are needed to validate the results and identify the conditions under which each threshold value yields the best MTV, ideally with an ex vivo validated volume segmentation.

### Disclosure

This work was supported by the Deutsche Forschungsgemeinschaft (German Research Foundation, Germany’s Excellence Strategy, EXC2180-390900677). We acknowledge support from the Open Access Publication Fund of the University Tuebingen. N.T. received personal fees from Novartis outside the submitted work and was supported by the Clinician Scientist Program Tuebingen (525-0-0). An ongoing research collaboration exists between N.T. and C.l.F. with ABX. C.l.F. discloses research grant from Siemens Healthineers, Telix, and reports consulting fees from Siemens Healthineers, Novartis, Telix, Astra Zeneca and Bayer. No other potential conflicts of interest related to this article were reported. 

## Supplementary Information

Below is the link to the electronic supplementary material.

Supplementary Material 1

## Data Availability

The datasets generated during and/or analyzed during the current study are available from the corresponding author on reasonable request.

## Abbreviations

CC1: Cell conditioner 1
CDKN2A/B: Cyclin-dependent kinase inhibitor 2 A/B
CT: Computed tomography
EANO: European Association of Neuro-Oncology
EMA: European Medicines Agency
FDA: US Food and Drug Administration
[^18^F]SiTATE: [^18^F]SiFAlin-TATE
[^68^Ga]Ga-DOTATOC: [^68^Ga]Ga-DOTA-Tyr3 octreotide
[^68^Ga]Ga-DOTATATE: [^68^Ga]Ga-DOTA-d-Phe1-Tyr3 octreotide
MRI: Magnetic resonance imaging
NF2: Neurofibromatosis type 2
PET: Positron emission tomography
SSAs: Somatostatin analogs
SSTR: Somatostatin receptor
SSTR2-IRS: SSTR2-immune-reactivity-score
SUV: Standardized uptake value
TBPR: Tumor-to-blood pool ratio
TERTp: Telomerase reverse transcriptase promoter
VOI: Volume of interest
