# Supplementary material for: Correlation of [18F]SiTATE-PET-tracer with somatostatin receptor expression in meningiomas: an exploratory ex vivo histological pilot study
Source: EJNMMI Res. 2026 Sep 4;16:134. doi: 10.1186/s13550-026-01505-w (PMC13542035; doi:10.1186/s13550-026-01505-w)
Supplement: Supplementary file 1 [file 13550_2026_1505_MOESM1_ESM.docx]

| Appendix  Supplementary Table 1: MTV vs MRI-volume | |
| --- | --- |
| Patient Nr. 1 |  |
| Volume meningioma (PET vs MRI in mL) |  |
| 50% isocontour | 1.10 vs 4.86 |
| SUV_min_ 4 | 0.02 vs 4.86 |
| SUV_max_ | 4.1 |
| Patient Nr. 2 |  |
| Volume meningioma (PET vs MRI in mL) |  |
| 50% isocontour | 16.48 vs 20.77 |
| SUV_min_ 4 | 24.46 vs 20.77 |
| SUV_max_ | 15.4 |
| Patient Nr. 3 |  |
| Volume meningioma 1 (PET vs MRI in mL) |  |
| 50% isocontour | 20.05 vs 24.15 |
| SUV_min_ 4 | 0.04 vs 24.15 |
| SUV_max_ | 4.2 |
| Volume meningioma 2 (PET vs MRI in mL) |  |
| 50% isocontour | 4.16 vs 4.86 |
| SUV_min_ 4 | * SUV_max_ < 4* vs 4.86 |
| SUV_max_ | 3.3 |
| Patient Nr. 4 |  |
| Not visible on MRI, detected on PET (n) | 1 |
| Volume meningioma (PET vs MRI in mL) |  |
| 50% isocontour | 1.99 vs 2.08 |
| SUV_min_ 4 | 6.43 vs 2.08 |
| SUV_max_ | 17.3 |
| Patient Nr. 5 |  |
| Not visible on MRI, detected on PET (n) | 1 |
| Volume meningioma 1 (PET vs MRI in mL) |  |
| 50% isocontour | 0.78 vs 0.80 |
| SUV_min_ 4 | 1.38 vs 0.80 |
| SUV_max_ | 12.0 |
| Volume meningioma 2 (PET vs MRI in mL) |  |
| 50% isocontour | 5.04 vs 5.22 |
| SUV_min_ 4 | 4.66 vs 5.22 |
| SUV_max_ | 7.6 |
| Patient Nr. 6 |  |
| Not visible on MRI, detected on PET (n) | 1 |
| Volume meningioma (PET vs MRI in mL) |  |
| 50% isocontour | 0.65 vs 0.81 |
| SUV_min_ 4 | 1.17 vs 0.81 |
| SUV_max_ | 11.7 |
| Patient Nr. 7 |  |
| Volume meningioma (PET vs MRI in mL) |  |
| 50% isocontour | 2.87 vs 1.16 |
| SUV_min_ 4 | 0.54 vs 1.16 |
| SUV_max_ | 5.2 |
| Patient Nr. 8 |  |
| Volume meningioma (PET vs MRI in mL) |  |
| 50% isocontour | 2.72 vs 47.51 |
| SUV_min_ 4 | 31.60 vs 47.51 |
| SUV_max_ | 19.1 |
| Patient Nr. 9 |  |
| Volume meningioma (PET vs MRI in mL) |  |
| 50% isocontour | 17.02 vs 29.15 |
| SUV_min_ 4 | 32.26 vs 29.15 |
| SUV_max_ | 15.1 |
| Patient Nr. 10 |  |
| Volume meningioma (PET vs MRI in mL) |  |
| 50% isocontour | 0.24 vs 0.34 |
| SUV_min_ 4 | 0.15 vs 0.34 |
| SUV_max_ | 6.7 |
| Patient Nr. 11 |  |
| Not visible on MRI, detected on PET (n) | 2 |
| Volume meningioma 1 (PET vs MRI in mL) |  |
| 50% isocontour | 1.43 vs 1.38 |
| SUV_min_ 4 | 2.77 vs 1.38 |
| SUV_max_ | 11.9 |
| Volume meningioma 2 (PET vs MRI in mL) |  |
| 50% isocontour | 0.58 vs 0.20 |
| SUV_min_ 4 | 0.39 vs 0.20 |
| SUV_max_ | 7.2 |
| Volume meningioma 3 (PET vs MRI in mL) |  |
| 50% isocontour | 0.37 vs 0.13 |
| SUV_min_ 4 | 0.40 vs 0.13 |
| SUV_max_ | 8.4 |
| Volume meningioma 4 (PET vs MRI in mL) |  |
| 50% isocontour | 1.12 vs 1.12 |
| SUV_min_ 4 | 2.05 vs 1.12 |
| SUV_max_ | 10.9 |
| Volume meningioma 5 (PET vs MRI in mL) |  |
| 50% isocontour | 1.65 vs 0.03 |
| SUV_min_ 4 | 5.42 vs 0.03 |
| SUV_max_ | 8.7 |
| Volume meningioma 6 (PET vs MRI in mL) |  |
| 50% isocontour | 0.89 vs 0.47 |
| SUV_min_ 4 | * SUV_max_ < 4* vs 0.47 |
| SUV_max_ | 3.5 |
| Patient Nr. 12 | Not evaluable in MRI due to artifacts |

| Supplementary Table 2: non-parametric vs. parametric test (in the case of a normal distribution) | |
| --- | --- |
| SUV_mean_ and IRS | |
| Pearson-Correlation |  |
| 50% isocontour (r; [95%-CI]; p) | 0.80; [0.42 - 0.94]; 0.002 |
| SUV_min_ 4 (r; [95%-CI]; p) | 0.797; [0.41 - 0.94]; 0.002 |
| Spearman-Correlation |  |
| 50% isocontour (r; [95%-CI]; p) | 0.82; [0.46 - 0.95]; 0.002 |
| SUV_min_ 4 (r; [95%-CI]; p) | 0.81; [0.43 - 0.95]; 0.002 |
| TBPR and IRS |  |
| Pearson-Correlation |  |
| 50% isocontour (r; [95%-CI]; p) | 0.78; [0.37 - 0.93]; 0.003 |
| SUV_min_ 4 (r; [95%-CI]; p) | 0.66; [0.14 - 0.89]; 0.02 |
| Spearman-Correlation |  |
| 50% isocontour (r; [95%-CI]; p) | 0.83; [0.49 - 0.95]; 0.001 |
| SUV_min_ 4 (r; [95%-CI]; p) | 0.72; [0.23 - 0.92]; 0.01 |
| SUV 50% isocontour vs SUV_min_ 4 |  |
| t-test |  |
| Difference; [95%-CI]; p | 0.63; [-0.37 - 1.64]; 0.19 |
| Wilcoxon test |  |
| Difference; [95%-CI]; p | 0.88; [-1.06 - 1.75]; 0.18 |
| SUV_mean_ depending on high and low SSTR-expressing groups |  |
| t-test |  |
| 50% isocontour  (difference; [95%-CI]; p) | 5.11; [1.79 - 8.44]; 0.006 |
| SUV_min_ 4  (difference; [95%-CI]; p) | 2.60; [0.74 - 4.45]; 0.01 |
| Mann-Whitney U test |  |
| 50% isocontour  (ΔHL difference; [96.36%-CI]; p) | 5.20; [1.65 - 8.38]; 0.009 |
| SUV_min_ 4  (ΔHL difference; [96.36%-CI]; p) | 2.42; [0.90 - 5.01]; 0.009 |

Supplementary Figure 1:

Supplementary Figure 1: Preoperative MRI (T1-weighted, post-contrast) of patient 11 is shown on the right, followed by postoperative MRI (T1-weighted, post-contrast, center right), PET (center left), and fused PET/MRI images (left). Red arrows indicate the meningioma components. The volume of interest (VOI) used for tracer uptake analysis is placed around an R2 residual tumor adjacent to the resected meningioma and demonstrating similar MRI morphological characteristics.
